# A single antenatal course of betamethasone adversely affects glucose regulation in adulthood and the next generation in childhood

**DOI:** 10.1186/1687-9856-2015-S1-O22

**Published:** 2015-04-28

**Authors:** Sarah Mathai, José Derraik, Wayne Cutfield, Stuart Dalziel, Janene Biggs, Craig Jefferies, Jane Harding, Paul Hofman

**Affiliations:** 1 Liggins Institute, University of Auckland, Auckland, New Zealand

## Objective

To assess whether a single antenatal course of betamethasone affects insulin sensitivity and other metabolic parameters in the offspring, and whether effects are transmitted to the next generation.

## Methods

A cohort of 52 adults (aged 35.7 years, 46% men, 23 born after steroid treatment) and their term-born children (n=61, aged 8.0 years, 52% boys, 49% from a parent born after steroid treatment), was recruited in Auckland. Insulin sensitivity and secretion were assessed using hyperglycaemic clamps in adults, and HOMA-IR in children. Other assessments included DXA-derived body composition, lipid profile, adipokines, and 24-hour ambulatory blood pressure monitoring.

## Results

Insulin sensitivity over the last 60 minutes of the hyperglycaemic clamp was 31% lower in the Steroid group (p=0.048), with a similar trend for overall insulin sensitivity (p=0.061). Steroid adults had a compensatory increase in first-phase insulin that was 53% higher than in controls (p=0.031), with total insulin secretion 44% higher in the Steroid group (p=0.044). Children of parents born after steroid treatment had higher fasting glucose (p=0.049) and insulin (p=0.008) concentrations than controls. HOMA-IR values indicated that children in the Steroid group were more insulin resistant than controls (p=0.006).

**Table 1:** Study outcomes in the offspring (adults, F1) born from mothers who were either treated with antenatal betamethasone (Steroid) or not treated (Control), and in subsequent generation (children, F2). Data are means and 95% CI, adjusted for confounders.

		Steroid	Control	p-value
Adults (F1)	n	23	29	
	1^st^ phase insulin (mU/l)	43.1 (30.9–60.2)	28.1 (20.9–37.8)	0.031
	2^nd^ phase insulin (mU/l)	57.5 (42.6–77.8)	41.6 (31.8–54.4)	0.068
	Total insulin (mU/l)	101.7 (74.7–138.4)	70.4 (53.5–92.6)	0.044
	Insulin sensitivity	16.1 (11.0–22.4)	23.1 (17.3–30.2)	0.061
	Insulin sensitivity last 60 min	14.3 (9.9–19.7)	20.6 (15.7–26.6)	0.048

Children (F2)	n	30	31	
	Fasting glucose (mg/dl)	4.88 (4.69–5.12)	4.67 (4.50–4.85)	0.049
	Fasting insulin (mU/l)	5.05 (3.98–6.41)	3.57 (2.79–4.58)	0.008
	HOMA-IR	1.10 (0.85–1.43)	0.74 (0.57–0.98)	0.006

## Conclusion

This study shows that maternal treatment with a single dose of betamethasone is associated with reduced insulin sensitivity in the offspring in mid-adulthood. Importantly, there is indication of an inter-generational effect, with the subsequent generation displaying increased insulin resistance.

